# Supramolecular assembly of protein building blocks: from folding to function

**DOI:** 10.1186/s40580-021-00294-3

**Published:** 2022-01-13

**Authors:** Nam Hyeong Kim, Hojae Choi, Zafar Muhammad Shahzad, Heesoo Ki, Jaekyoung Lee, Heeyeop Chae, Yong Ho Kim

**Affiliations:** 1 SKKU Advanced Institute of Nanotechnology (SAINT), Sungkyunkwan University, Suwon, 16419 Republic of Korea; 2 School of Chemical Engineering, Sungkyunkwan University, Suwon, 16419 Republic of Korea; 3 Department of Nano Engineering, Sungkyunkwan University, Suwon, 16419 Republic of Korea; 4 Center for Neuroscience Imaging Research, Institute for Basic Science (IBS), Suwon, 16419 Republic of Korea

**Keywords:** Supramolecular assembly, Protein design, Protein folding, Protein–protein interaction

## Abstract

Several phenomena occurring throughout the life of living things start and end with proteins. Various proteins form one complex structure to control detailed reactions. In contrast, one protein forms various structures and implements other biological phenomena depending on the situation. The basic principle that forms these hierarchical structures is protein self-assembly. A single building block is sufficient to create homogeneous structures with complex shapes, such as rings, filaments, or containers. These assemblies are widely used in biology as they enable multivalent binding, ultra-sensitive regulation, and compartmentalization. Moreover, with advances in the computational design of protein folding and protein–protein interfaces, considerable progress has recently been made in the de novo design of protein assemblies. Our review presents a description of the components of supramolecular protein assembly and their application in understanding biological phenomena to therapeutics.

## Introduction

**Fig. 1:**
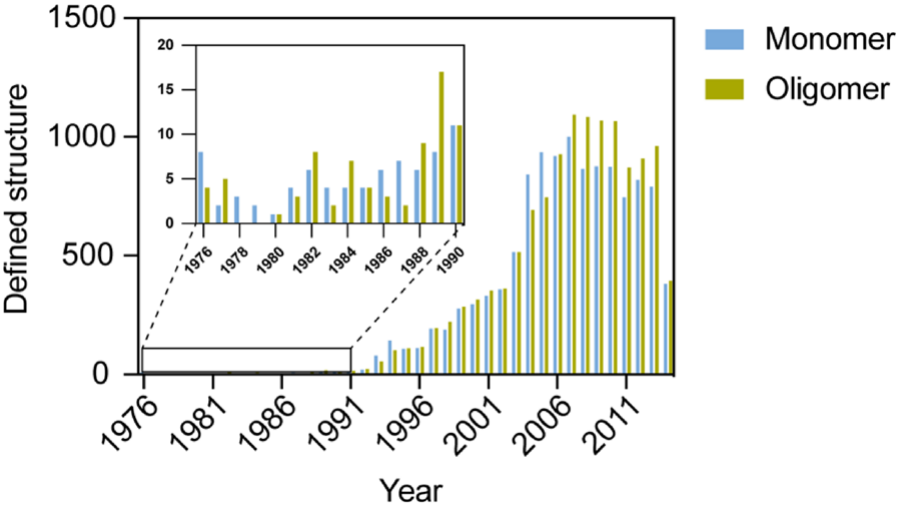
Statistical analysis of annually defined structure in Protein Data Bank (PDB) (Data obtained from https://www.rcsb.org/stats)

Supramolecular assembly is a very common phenomena in nature and these natural supramolecular proteins have various structures, from simple structures to complex structures [1, 2]. As the increasing development of structural analysis, such as Cryo-EM, it is possible to observe the conformation of supramolecular assembled structure [3]. Before the 1990s, protein structures were scarcely elucidated; however, more protein structures have been identified by advanced technology (Fig. 1). More structures emerge from oligomeric proteins than monomeric proteins, providing us more opportunities to study protein assembly [3]. In this paper, we will discuss about the component of supramolecular protein assembly and classify their conformation according to structural dimensionality. Lastly, we speculate the biological phenomena from organism and their application with their own properties.

## The component of protein assembly

**Fig. 2:**
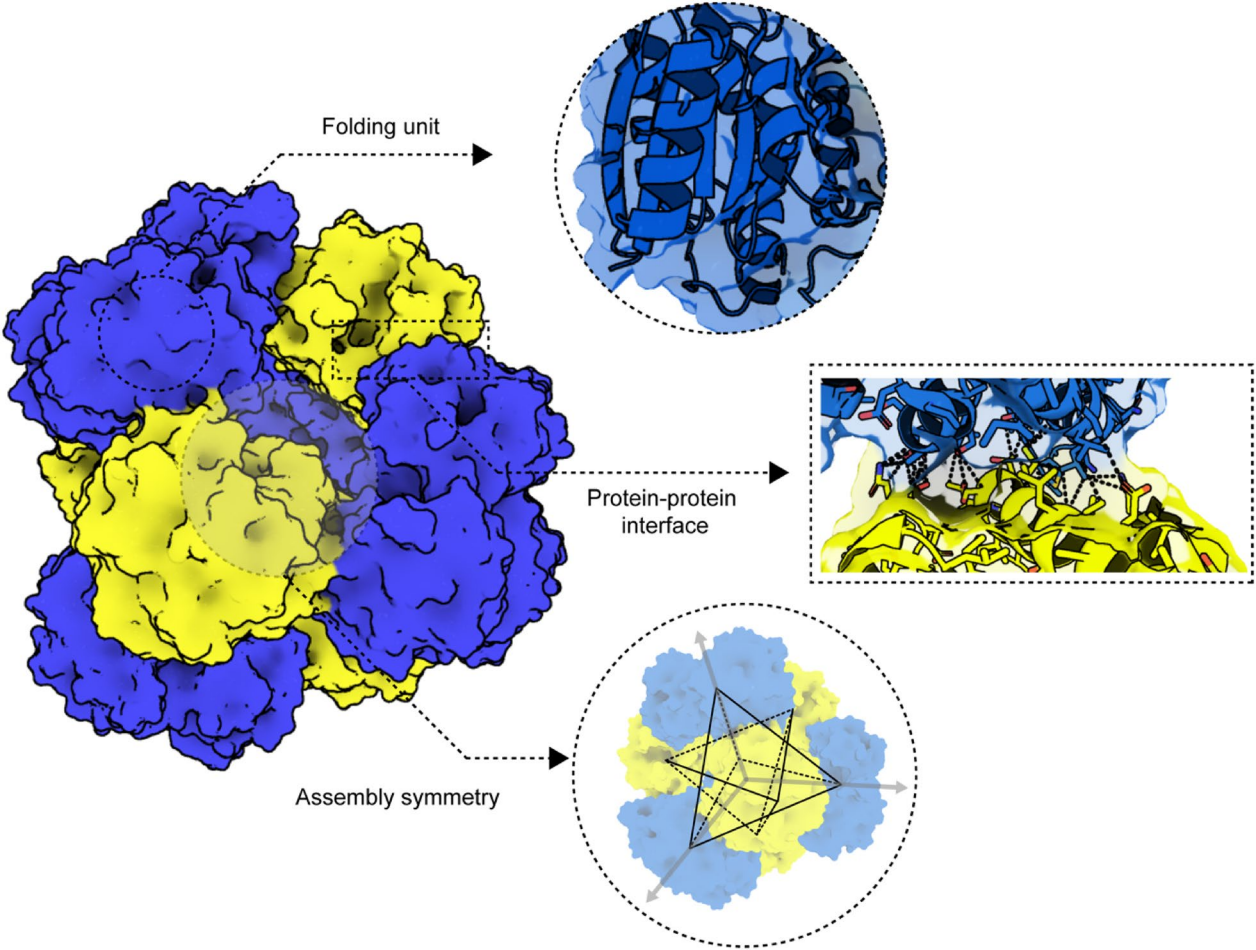
Schematic image for description of three components of supramolecular protein assembly

We define the components of protein assembly in three aspects: folding structure unit, protein–protein interface, and assembly symmetry (Fig. 2). Each components determine the overall structure of supramolecular protein structure and their functions.

### Folding structure unit 

**Fig. 3:**
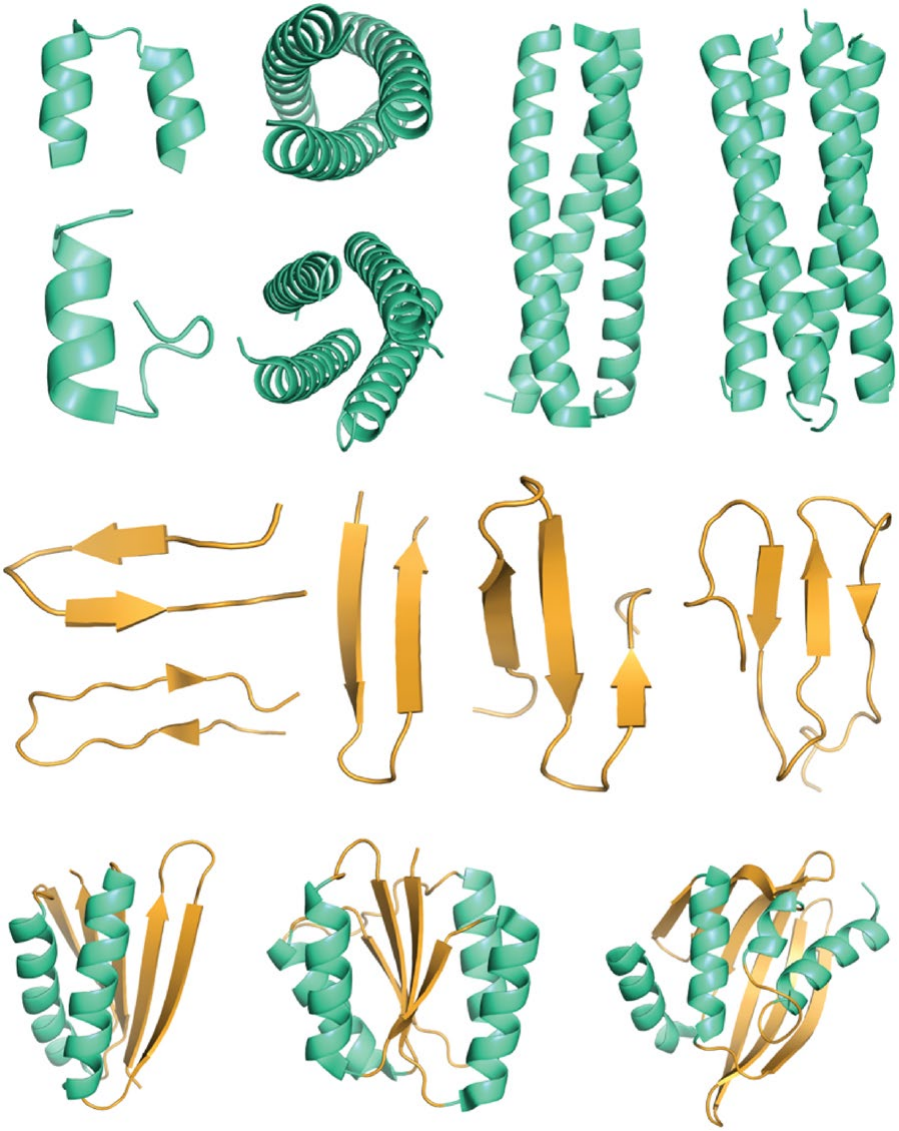
Representative example of folding unit: α-helix, β-sheet, and α/β mixed structure

The folding unit is the basic building block for supramolecular protein assembly [4]. These building blocks are mostly composed of α-helices, β-sheets, or a mixture of them (Fig. 3A). The symmetry of the units and dimensions of the supramolecular protein are determined, depending on how the folding unit is configured. A better understanding of the proteins contributes to designing new supramolecular structures from rational design to de novo design. Rational design is inspired by common features and motifs from existing proteins [5, 6]. The α-helical structure is well characterized by Crick parameterization, and the sequence pattern of heptad repeats has been widely studied [7]. This enables the rational design of the alpha helical coiled-coil motif. Recently, the de novo design of proteins that start from scratch can create a new topology beyond experimentally determined structures. De novo design generates a building block based on the basic physical principle of protein by using computational power. There are many open-source programs that can help us build α-helices or β-sheets and complex forms of building units. To build a coiled-coil motif, we can easily build unit structures from CCCP (Coiled-coil Crick Parameterization) [7], CCBuilder2.0 [8] This allows researchers to build generalized models of coiled coils based on the Crick parameters or to calculate the folding stability of the resulting coiled coils [9]. The design of the β-sheet structure has non-local interactions where more β-sheet fractions give some sequence distance between each β-sheet unit, leading to slower folding rate [10] or misfolding [11, 12]. So, we need to predict and calculate the proper distance for every different scaffold of β-sheet structures. ‘BluePrintBDR’ mover in rosetta makes it easier to create a new topology structure based on the 2D map indicating which residue is pairing with a particular residue. The development of computational power and increased protein structure databases has expanded our knowledge to understand and design folding units.

### Protein–protein interface

The protein interface is a major determinant of the structure and function of natural proteins [13]. In nature, the signal transduction pathway is determined very precisely according to the difference in the protein interface of each ligand at the binding site of the multiligand receptor [14]. Among them, the supramolecular building block determines the binding stoichiometry between unit structures and controls the overall morphology according to the energetic favorability of each facet. The protein interface is delicately determined according to the size of the exposed solvent-accessible area [15, 16], hydrophobic packing [17, 18], and the existence of hot-spot residues [19, 20]. It can be classified into three categories based on importance and structure: helix interface, beta-sheet elongation, and metal coordinates.

#### Helical interface

The helical interface is the most common structure of the protein interface [21–24]. It is used in various structural regulations, from the very small protein assembly structure of the coiled-coil to heterogenous protein assembly, such as bacteriophage virion coating. The helix structure appears to be a simple helical structure; however, it is based on how each helix structure is connected in the helix-turn-helix structure and new interface such as parallel/antiparallel alignment can be created.

Amphiphilic interfaces of the helix are important for designating the directionality of the assembly. Hydrophobic patches at the protein–protein interface allow the monomers to form stable complexes [25]. In contrast, unfavorable buried polar residues destabilize the bonding interface [26]. Therefore, modulating the amphiphilic interface, which includes both hydrophobic and hydrophilic interactions, is a key component of protein assembly.

#### Beta-strand elongation

The beta-strand interface, which intermolecular network of hydrogen bonds, are widely occur in protein assembly. With the development of protein structure analysis technology, the morphology of the beta-sheet fiber structure of various structures was discovered and classified according to the beta strand elongation direction (parallel/antiparallel) and the symmetry of the steric zippers forming the core of the fiber structure [27]. The interface of beta-strand used to design many artificial amyloid-like structures assembled in the form of fibrils [28, 29] and gel [30, 31] and tandem repeat structures of ring or pore shape. The design of the beta-strand interface is very complex because the sheets bond intermolecularly and do not elongate except when the beta-strand is fully aligned within an axis. Recently, Baker et al. demonstrated the first de novo designed beta-barrel structures by establishing the geometric parameters of antiparallel beta-barrels to build an ideal backbone template and assign residues for structural stabilization [32].

#### Metal coordinates

Metal coordination plays an essential role in developing unit protein structures for regulatory and enzymatic reactions, including metalloenzymes, chloroplasts, and zinc-finger families [33]. Metal coordinates that exist in nature are necessary for properly folding proteins and creating complex quaternary structures through the bridging of metal ions [34, 35]. As a representative example of metal coordination, zinc finger proteins utilize a coordination zinc ion to maintain the folded structure, which is important for recognizing target DNA for transcriptional regulation [36, 37]. In addition, since metal coordination reacts very sensitively to pH change, the assembly pattern is diversified according to the tissue environment. Salgado et al. suggested the concept of MeTIR, metal-templated interface redesign, which induces self-assembly of the supramolecular structure in the presence or absence of metals [35].

### Assembly symmetry

**Fig. 4:**
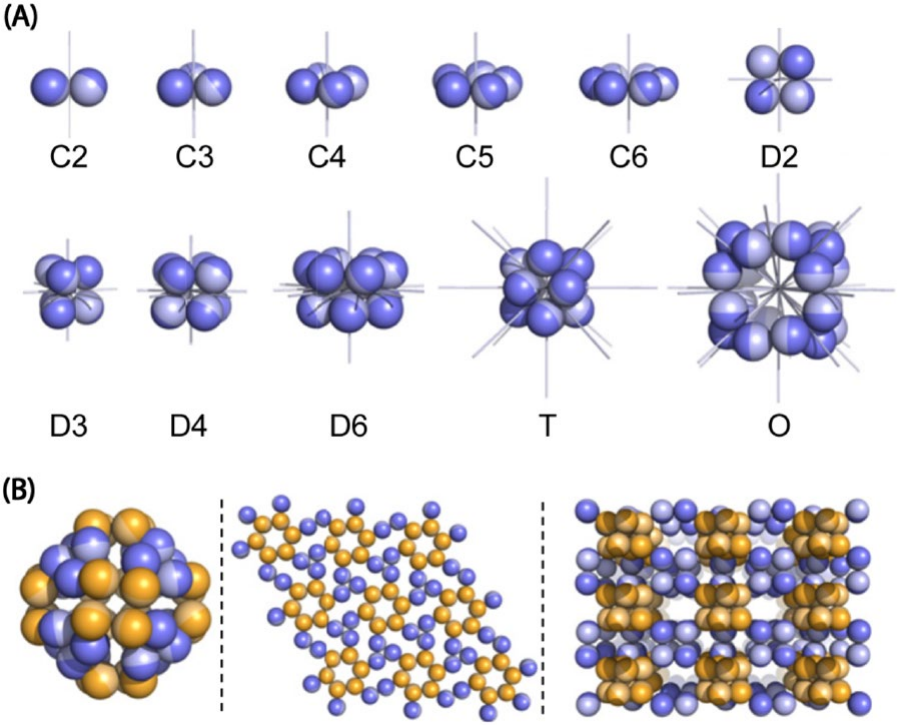
Schematic image of symmetric oligomeric building blocks and example two-component assembled structure. **A** Illustration of point group symmetries. **B** Representative example of octahedral symmetry constructed by combining C3 trimer and C4 tetramer (left), p6 symmetry constructed by combining C3 trimer and C6 hexamer (middle), and p422 symmetry assembled by combining D2 tetramer and D4 octamer (right) (Image reprinted with permission from Ref [38])

The overall assembly structure is strongly limited by the number of ways in which protein subunits may associate in three dimensions. Protein assembly can have various structures that can create a more complex and high-order hierarchical structure through a combination of the symmetry of the folding unit. For example, if a possible combination is predicted with two oligomeric components, each with C2 symmetry and D3 symmetry, a total of 11 candidates such as p312, P622, and P4132 are possible (Fig. 4) [38]. This combination modulates the overall size by controlling the number of folding units that determine the overall supramolecular structure according to the difference in the affinity of the interface. Moreover, an internally symmetric folding unit can designate the dimensions of the assembly pattern with open or closed symmetries. When the interface of the folding unit is open, because the unit is assembled in one direction, the entire structure is propagated in the form of a fibril or 2D sheet to form an assembled structure of various sizes. However, in the case of a closed interface, a cage or cyclic structure, in which the size of the entire structure is defined, is formed.

## Various structures of supramolecular protein assembly

**Fig. 5:**
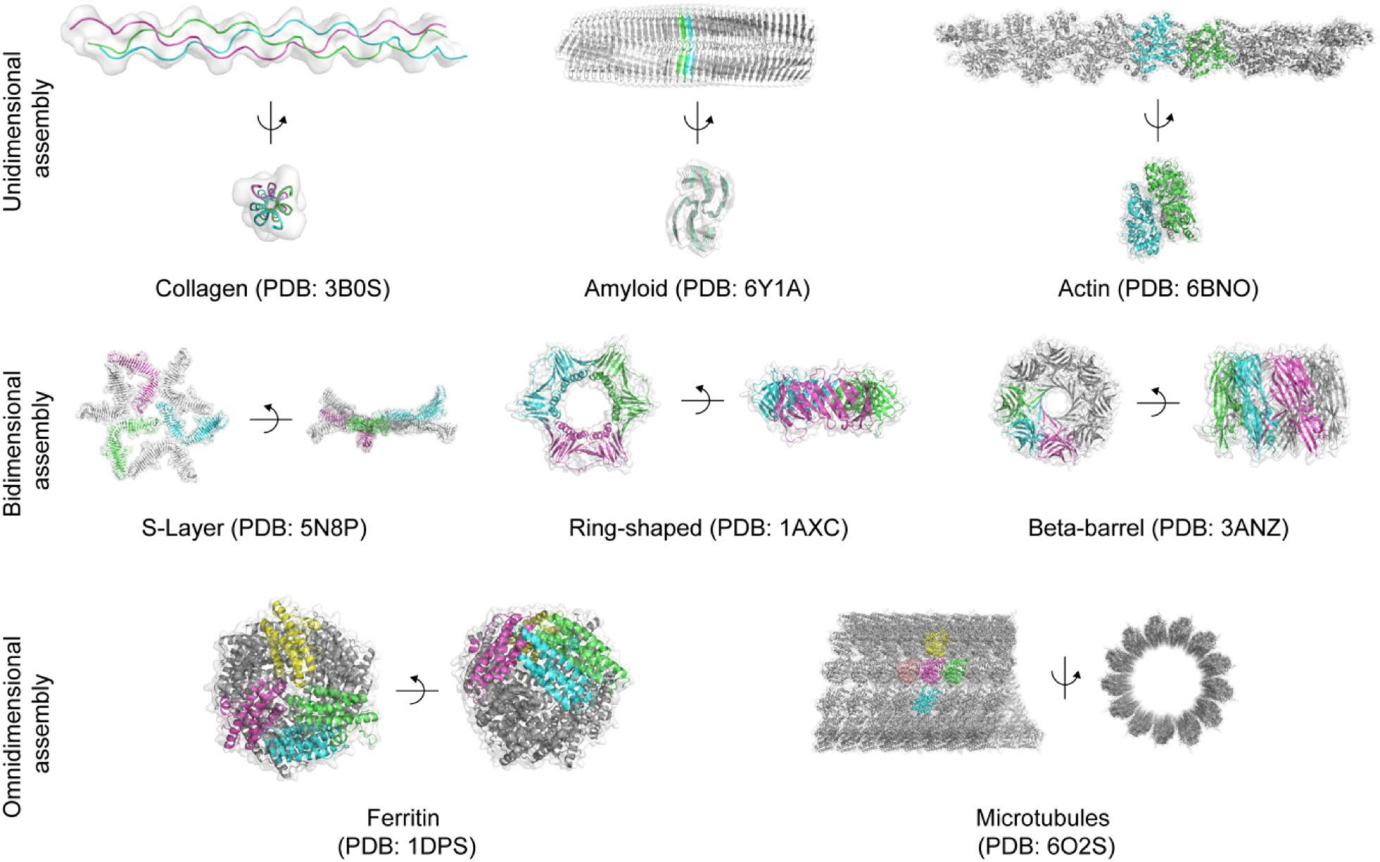
Various assembled structure and their folding unit of supramolecular protein assembly

Supramolecular protein assemblies can be classified according to their orientation along a dimensional axis (Fig. 5). Dimensions are defined in the direction of overall structural expansion as the folding structural units are assembled. Dimensions are classified according to the number of axes that contribute to the addition of units and expansion of the overall structure: Unidimensional assembly, in which units assemble along a single axis to elongate the protein, Bidimensional assembly, in which units join together along a dual axis to expand a protein, and omnidimensional assembly, in which units are assembled along three or more axes to extend a protein.

### Unidimensional assembly

Proteins with a unidimensional assembly are elongated as the unit structures are added along a single axis. Typical examples of unidimensional assembly are fiber structures, including collagen [39–43], amyloid [28, 29, 44–48], and actin filament [49–52]. Collagen is a linear triple-helix structured protein [39] that accounts for approximately 70% of the extracellular matrix dry weight; thus, it is the most abundant protein in mammals. The single left-handed polyproline II-type helix composed of the repetition of Xaa-Yaa-Gly assemble into triple helix bundle with right-handed conformation through the hydrogen bonds between neighboring strands, especially N–H(Gly)⋯O = C(Xaa), to bury Gly residues toward the core of the triple helix and expose the other residues to the outer surface. Amyloids are elongated, unbranched β-sheet fibrils that can cause various diseases. There are many different protein units that form amyloid structures, such as β-amyloid peptide(Aβ) [29, 46], α-synuclein [47], and tau protein [48]. Individual β-strands assemble through hydrogen bonds to generate β-sheets, and several β-sheets twist around a central axis, assembling through a steric zipper interface, which is a tight dehydrated packing between neighboring β-sheets. Actin filament (F-actin) is a two-long-pitch helically stranded microfilament composed of an actin monomer called G-actin, which is an important component of the cell cytoskeleton and muscle tissue [50, 51]. Sault bridges, geometric surface complementarity, and hydrophobic interactions on the interstrand and intrastrand interface, particularly the loop compartment on the interface, stabilize F-actin.

Unidimensional assembly proteins, such as fibrils and fibers, can be designed to be extended along a one-dimensional axis to compensate for the short length, low mechanical strength, and stability of self-assembled protein in nature. Nanofibers are designed to form a desired structure and assembly with unique properties only under intended conditions such as temperature and pH to have high utility and specificity; therefore, they can be utilized in various fields such as biomaterials and medicine. In addition, the information obtained in the design process can provide a chance to reveal the mechanisms and properties of self-assembled nanofibers that exist in nature like collagen [39–43], amyloid [28, 29, 44–48], and actin filament [49–52], previously described, which are difficult to understand only with structural information [53, 54]. Based on this information, self-assembled nanofibers composed of simple alpha helix [55–57] or beta-strand [58–64] can be designed. For example, Ronak et al. designed de novo three-stranded β-sheet nanofibrils (TSS1) composed of 29 amino acids, mostly lysine and valine for self-assembling mechanically rigid hydrogel fabrication [58]. Novel self-assembled nanofibers composed of complex structures that do not exist in nature can be designed over self-assembled nanofibers composed of simple alpha helices or beta sheets. Sabine et al. designed complex βαβ unit structures that self-assemble into fibers with an alpha helical outer surface by linking two β-fibrillizing peptides to the alpha helix for stabilization and easier functionalization of the nanofiber [53].

### Bidimensional assembly

Proteins with a bidimensional assembly are widened as unit structures are added along the two axes. One example of a bidimensional assembly is a sheet structure, including bacterial S-layers [65–70], which are two-dimensional arrays that coat the surface of bacteria or archaea [68]. S-layers are generated by the assembly of S-layer protein subunits through van der Waals force, ionic bonds, and hydrogen bonds [67], which develop various types of lattice structures from hexagonal (p3, p6), square (p4), and tilted (p1, p2) space groups [70].

The ring structure is another example of a bidimensional assembly, and it includes DNA clamps [71–73], helicases [74–77], nucleases [78–80], which are ring-shaped protein complexes that contribute to the metabolic process of DNA or RNA. DNA clamps are formed by the tight assembly of subunits that are composed of the α-helix and β-strand complex to generate a closed ring encircling DNA strand [71–73]. Subunits assemble into a ring structure with a positively charged inner surface; therefore, the ring can electrostatically interact with the negatively charged DNA strand [71]. In the case of helicase, four out of six superfamilies have a ring-shaped assembly structure [77], and they commonly take the form of a hexameric structure with a central pore. The core of the ring contains nucleotide binding sites between the subunits, which usually have an arginine finger that is involved in nucleoside triphosphate (NTP) binding and hydrolysis [74–77]. Several types of nucleases, including bacteriophage λ-exonuclease [78], *E. coli* RecE [79], and Cas4 nuclease SSO0001 [80], have a pore-containing toroid structure, and they exhibit diverse morphologies such as trimer [78], tetramer [79], and pentamer [80]. The assembly of these structures is mediated by subunit interfaces with hydrogen bonding, ion pairs, and hydrophobic interactions [79]. Some nucleases have unique funnel-shaped pores, which have a wide entrance and a narrow exit that can accept double-stranded DNA at the entrance and pass only the single-stranded DNA through exit [78, 79].

The other representative morphology of the bidimensional assembly is a beta-barrel structure. Beta barrel structures include β-barrel transmembrane proteins [81–85] and green fluorescence protein (GFP) [86–88]. Transmembrane proteins with β-barrel structure are located in the outer membranes of chloroplasts, gram-negative bacteria, and mitochondria [85], and most β-barrel transmembrane proteins are composed of an even number of strands arranged in anti-parallel [81–85]. β-barrel transmembrane proteins have abundant intrastrand hydrogen bond networks; therefore, they are stable in the membrane environment. GFP is a β-barrel protein that emits green fluorescence, and 11 strands of β-sheet form a cylindrical outer surface connected by short helical loop structures, protecting 3-amino acid fluorophores inside the barrel [86–88]. The polar interactions surrounding the central fluorophore mediate proton rearrangements, leading to the activation of GFP [87].

Through the two-dimensional protein array assembly design, proteins are repeated in a specific order. Thus, specific proteins designated by the tailor can be arranged in a constant valency and order. However, 2D protein arrays are very rare in nature, such as the exoskeleton of the surface layer of many bacteria; therefore, it is essential to design the properties of 2D protein arrays [89]. Modulating two docking axes and interfaces can determine the self-assembly mechanism and overall symmetry structure of bidimensional proteins and make them more diverse and controllable structural and functional 2D materials such as 2D arrays [90–93] that do not exist in nature. For example, Chen et al. designed 2D arrays of homodimer protein building blocks by modulating the 4-binding interface of the helix bundle following the C_12_ layer symmetry group [90].

The ring structure, which are bidimensionally assembled in a cyclic arrangement, is another representative example of a bidimensional assembly protein design. The protein pore size of the designed self-assembled nanoring can be controlled by the relationship between the oligomer state and pore diameter [94]. However, the de novo design of nanoring with large pores is challenging for thermodynamic stabilization because the large surface-area-to-volume ratio of the nanoring results in a low stabilizing interaction density [95]. The design of the alpha-helix ring structure [94–98] is simple owing to the specific parameter type of the backbone by the crick and versatility of the application using an outer alpha-helical surface. The beta barrel ring is very complicated because it tends to cause misfolding and aggregation easily if not properly controlled [32]. After developing structural understanding and computational design, the design of beta barrels to utilize rigid self-assembly ability has started [32, 99, 100]. In addition, through development of design method containing both alpha helix and beta sheets [101, 102], self-assembling proteins with both secondary structures can be designed for easier stabilization even with compact protein and expansion of design available protein structure pool. For example*, *Lim et al. increased the binding stability and strength of the unstable short alpha helix bundle by linking beta sheets as a self-assemble inducing segment with an alpha helix, which eventually forms a beta barrel inside the alpha ring [103].

### Omnidimensional assembly

Proteins with an omnidimensional assembly are expanded as the unit structures are added along three or more axes. Tube structures, examples of omnidimensional assemblies, include helical virus capsid [104–107] and microtubules [108, 109]. Helical viruses, represented by the mosaic virus family [104–107], are composed of helical nucleic acid coils and capsid proteins, where capsid protein subunits cover the nucleic acid. The assembly of the helical virus is mediated by subunit-RNA interactions and subunit-subunit charge interactions. An abundant salt bridge from intersubunit ion pairs stabilizes the interface between the subunits. In addition, the electrostatic interactions between the RNA backbone and positively charged part of the protein derive the subunit-RNA interaction. Microtubules are cellular structures that form the cytoskeleton of eukaryotic cells [108, 109], and α/β-tubulin dimers polymerize into a cylindrical complex in a specific direction, leading the microtubule to have a subunit-adding site and subunit-dissociation site. Subunits assemble in a way that enables the additional attachment of several microtubule-binding proteins by negatively charged the outer microtubule surface by placing the acidic residues on the subunit C-terminal tails [109].

Another typical example of omnidimensional assembly is a cage structure, including polyhedral virus capsid [110–113], ferritin [114–120], lumazine synthase [121, 122], vaults [123–126], clathrin lattice [127], and heat shock proteins [128–130]. Polyhedral viruses are composed of central nucleic acids and surrounding polyhedral capsids, whose subunits are assembled through subunit-subunit interactions and subunit-RNA interactions [110]. Subunit-subunit interactions originate from integrating electrostatic repulsions and hydrophobic attractions, where the subunit-RNA electrostatic interactions originate from the interface between the negative-charge RNA backbone and the positive-charge N-terminal of the subunits [110–113]. For the other examples of cage structure, there are various protein complexes that control the life activities of diverse organisms, by storing the specific target material [114–120], performing enzymatic activities [121, 122], mediating cellular processes [123–126], coating vesicles [127], and protecting cell components from the stressful environment [128–130]. Ferritin is a hollow cage structure composed of a four-helix bundle subunit assembly, containing sufficient metal-protein interactions to generate iron binding sites, since this protein structure encapsulates iron [114–120]. The lumazine synthase cage is generated from the assembly of the pentamer consisting of five subunits, which are built with several β-strands and α-helixes [121, 122] abundant hydrogen bonds and ionic contacts between subunits allow lumazine synthase to function as favorably binding inhibitors [122]. Vaults are created by assembling three subunit proteins, including the major vault protein (MVP), a subunit consisting of several β-strands [123]. Assembly of vaults is mainly derived by helix-helix interactions, with polar residues facing the surface of the whole structure and hydrophobic residues located on the interface between neighboring helixes [123–126]. The clathrin lattice is an assembly of the clathrin subunits, which can form various cage structures from small cages of 28 and 36 assembly units to hexagonal arrays and soccer ball structures. They are stabilized through fixed contact patterns adjacent to each end of the strand [127]. Heat shock proteins, particularly chaperonin (HSP60), are another representative example of cage structure assembly. Chaperonins consist of two consecutive stacked rings composed of seven, eight, or nine subunits [128–130]. The inter-ring interface, which consists of electrostatic interactions between the positively charged residue in one ring and the negatively charged residue of the other ring, provides the structural basis of chaperonins [129].

In addition to the structural limitations of nature-derived self-assembled hierarchical nanostructures that have not suitable valency or size for various applications, researchers have designed highly ordered de novo supramolecular omnidimensional assembly structures such as nanotubes [55, 91, 131–134] and nanocage [135–143] with variable size, shape, and symmetry using computational tools. Designing omidimensional self-assembly structures with regularly repeating subunits in tailor-defined constant valency makes them suitable for use in various fields through interior or exterior surface functionalization. The subunit structure design of hierarchical nanostructures determines the overall size and shape of self-assembled nanostructures, such as the subunits per helical turn, helical pitch, and pore size of nanotubes, or the high-order symmetry of nanocages such as dihedral, tetrahedral, octahedral, and icosahedral. In designing a self-assembling nanocage, combining two types of symmetrical subunits can create nanocage structures with diverse high-order symmetry, size, and valency. [136–141, 144]. However, even if the same symmetric building blocks are used, they can be assembled into nanocages of different high-order structures with different valencies, depending on which symmetry axis alignment the blocks are arranged. For example, C3 and C2 building blocks with tetrahedral or icosahedral axes assembled in tetrahedral and icosahedral nanocages, respectively [137, 138]. In a different way, a single type of symmetrical subunit can also form a hierarchical supramolecular nanocage alone by designing the symmetric building block unit interface to self-assemble each other that does not participate in the previous assembly. Using this method, placing trimer proteins as C3 symmetry building blocks to three-fold rotational symmetry axes of tetrahedral or octahedral point group symmetry can form supramolecular nanocages with only one new interface design between the trimeric building blocks for self-assembly [140, 142].

## Biology of supramolecular protein assembly

Naturally derived protein self-assembly or rationally designed folding units have shown the strength to construct various sophisticated protein nanostructures. It can produce enormous properties that cannot be created within a single monomeric protein conformer. Nature utilizes protein assembly to build complex biological phenomena such as signal transduction, cell growth, and immunology. Furthermore, protein assemblies can be applied as templates for development from functional biomaterials such as biomimetic materials to drug delivery platforms, biomedical diagnostics, and therapeutic platforms including vaccines. Here, we classify representative biology of supramolecular protein assemblies, such as matrix scaffolds of cell growth, encapsulation of functional cargo inside supramolecular structures, and hybridize designed proteins with unprecedented materials.

### Matrix scaffold in cell growth

The nature-derived self-assembly of various proteins and peptides, such as collagens, proteoglycans, laminins, and fibronectin form the extracellular matrix (ECM) of each tissue, and their association determines the structure, function, and organization of the tissue. Hence, elucidating the mimic of ECM-derived biomolecules is an important aspect of cell biology and growth [145–148]. The utilization of naturally derived ECM as a cell culture substrate has become a viable option for cell growth. To this end, the self-assembly properties of various peptides, such as RAD16 [149–151], amyloids [152–154], have been utilized as substrates to control cell growth. Furthermore, ECM based on various adhesion proteins, such as collagen [155], fibronectin [156], and laminin [157] has been utilized as a scaffold for cell growth [158]. These proteins utilize cell-binding epitopes for binding to integrins, which consists of an adhesion protein derived small peptide sequence. For instance, the peptide sequence derived from collagen, fibronectin and laminin is RGD, RGDS and IKVAV/YIGSR, respectively. [159]. In addition, the self-assembly property of silk like protein (FN-silk from fibronectin) to form networks of microfibers has been utilized as an ECM. This association provides a three-dimensional (3D) microfiber network with specific sites for cell anchorage, which makes the cells remain viable for more than 90 days [160]. In addition, the spontaneous self-association of peptides can be utilized for cell growth facilities by employing matrix-like platforms. Particularly, the peptides with elongated hydrophobic hydrocarbon chains (HHC) or having hydrophilic groups on ends, -self-assembled with—external hydrophilic surfaces. These peptides, known as bolaamphiphile peptides, have a higher propensity to form flat layers upon self-assembly, making them a good candidate for working as substrates [161–163]. Recently, da Silva et al. utilized the self-assembly property of a bolaamphiphile peptide which form well defined nanosheets in water via association of the peptide backbone. The assembly is driven by the nucleation phase and serves as a substrate for cell growth matrix, which—proved its worth with human corneal stromal fibroblast (hCSF) cell growth [164]. On the other hand, the self-assembly property of laminin-derived peptides, triggered by slight hydrophobic modification, has been utilized for the formation of supramolecular structures suitable for cell growth applications [165–167]. To this field, most recently, Jain et al. controlled neuronal cell growth by a peptide-based supramolecular self-assembled matrix. The developed supramolecular gel was composed of self-assembly of two peptides (IKVAV and YIGSR), which facilitated the interaction between cells and the matrix. Their developed gel not only proved its worth by controlling neuronal cell growth and SHSY5Y neuroblastoma cells but also the proliferation in C6-glial cells [168]. In the field of gel-based matrices, another group of scientists made hydrogels from self-assembled peptides [167, 169, 170]. Recently, Aye et al*.* proposed the use of peptide-based hydrogels as biocompatible scaffolds for regenerative medicine. They developed a hydrogel composed of biomimetic inclusion of three different peptide sequences. During the in-vitro testing with human mammary fibroblasts cell culture, the hydrogel proved its worth by improved cell adhesion, growth, and proliferation [171]. Meanwhile, a group of scientists have explored the self-assembly of a set of dipeptides (X-ΔPhe) containing ΔPhe (α,β-dehydrophenylalanine) at their C-terminus and their possible use in drug delivery [172–175]. Inspired by the self-assembly property of dipeptides, Yadav et al. utilized a 3D platform, composed of dipeptide‑based hydrogel [174] as a scaffold for three dimensional cell growth. They utilized a leucine-α,β-dihydrophenylalanine (Leu-ΔPhe)-based hydrogel capable of forming hydrogels in the MPa range required for bone-like engineering stiffness at relatively low concentrations. Their results indicated the healthy condition of cells with good functionality, making Leu-Δphe hydrogel a suitable dipeptide candidate for three dimensional scaffold for cell culture [176].

### Membrane associated protein assembly

Membrane proteins are proteins that span the cell membrane and play important roles in controlling cellular behavior, including selective molecular transport through the bilayer, nutrient uptake, and signal transduction [177, 178]. In particular, the transmembrane domain regulates cellular activity by organizing oligomers with specific symmetry. Most transmembrane domains have a α-helix structure and are reverse amphiphilic, with hydrophilic surfaces facing inward and hydrophobic surfaces facing outward. There were many attempts to mimic and engineer the nature-derived transmembrane protein, such as exporting key motifs of *E.coli* Wza to conduct ion and bind blocker [97], deleting specific domain of FhuA to conduct ion [179], attaching ligands on Salmonella typhi ClyA to selectively shuttle the large analyte proteins[180] or DNA molecules [181], and adding subunits of ClyA to vary the size of pore [182].

The de novo design can artificially build up the transmembrane protein with desired function or structure that is hardly found in nature. 12-helical potassium conducting protein that performs superior conductance of K^+^ over CH_3_NH^3+^, Cs^+^, Na^+^, Ba^2+^ is computationally designed and inserted into membrane through repositioning hydrophobic residues [95], and four-helical bundle containing two di-metal binding sites that selectively passes Zn^2+^ and Co^2+^ is reported [183]. The structure of concentric ring [95] and polar ionizable ligand containing complex [183] can be further utilized as a platform to selectively conduct various target ions, in ways that are not possible with native channels. The study of transmembrane pore that is large enough to pass the small molecule [95] can be further applicated in small molecule delivery system, which is attractive in medical and pharmaceutical fields. Furthermore, by computationally designing combinations of non-natural iron diphenylporphyrins and D2-symmetric four-helix bundles, it is possible to mimic the transmembrane electron transportation, a key component of ATP production and photosynthesis. This unique metaloprotein complex successfully transported electrons, which opens up the possibility of further designing artificial photosystems [96].

### Nano-cage structure for surface displaying epitopes, encapsulation and cell signaling

Nanocage structures can be used for displaying epitopes, encapsulation, and cell signaling using multivalency, highly ordered repetitive arrays, and hollow structures. Multivalency and highly ordered repetitive arrays of clustered epitopes displayed on the surface of self-assembled nanostructures can increase avidity compared to single epitopes alone [184–189]. In addition, multiple types of surface functionalization units attached to the surface allow supramolecular structures to become multifunctional nanocarriers [190]. In addition, the hollow structure of the hierarchical structure reassembly and disassembly under specific conditions by subunit interface interactions can be used to encapsulate drugs or genes that make nanocages an effective delivery platform [191, 192].

**Fig. 6:**
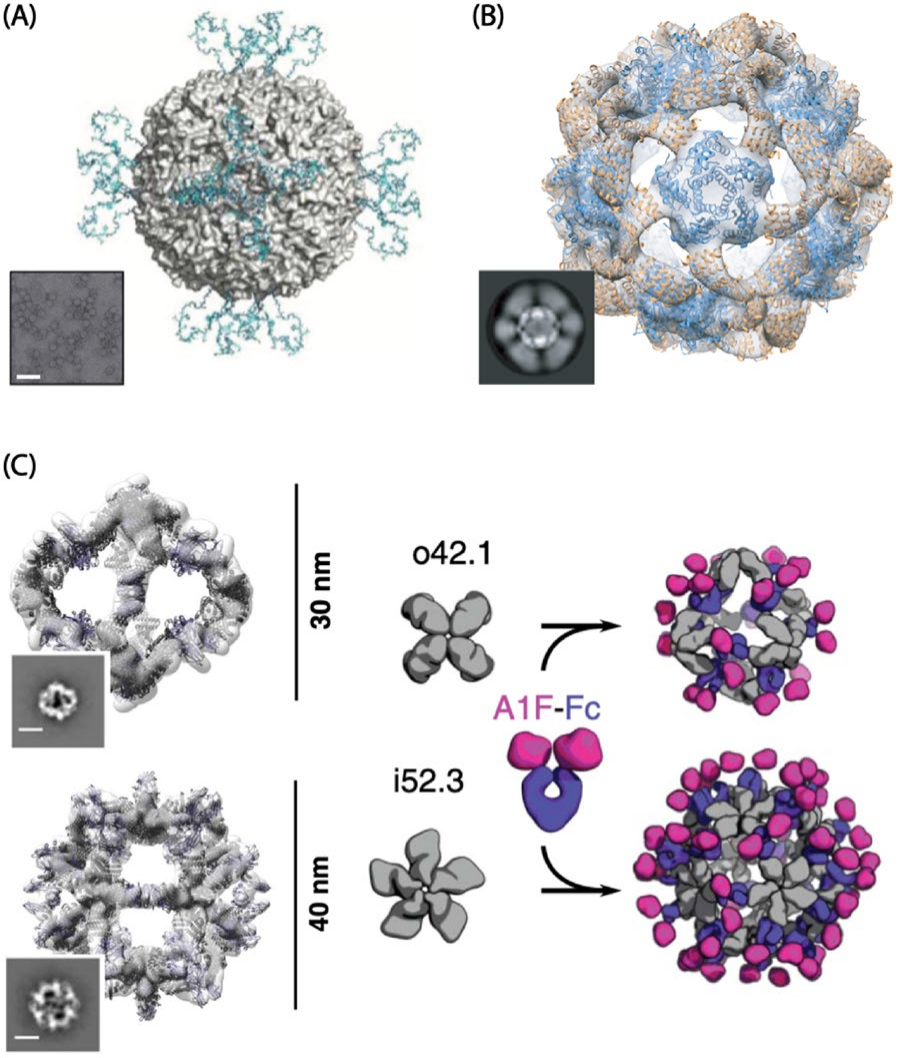
Schematic image of application of a nanocage. **A** The ferritin nanocages displaying EV71 antigens on the surface as epitopes for the use of vaccine. **B** The BG505 SOSIP displaying de novo designed icosahedral nanocage. **C** The octahedral (up) and icosahedral (down) antibody nanocages with fusion of angiopoietin-1 F-domain (A1F) and Fc (Image reprinted with permission from Ref [135, 196, 203])

Naturally derived self-assembly proteins such as ferritin and lumazine synthase have been used for cell signaling [186] and display of epitopes (antigens) from influenza [193], HIV-1 [194], Epstein-Barr virus [195], SARS-CoV-2 spike protein [188, 189], and Enterovirus 71 (EV71) [196] for immune response activation due to their biocompatibility, stability, and easy modification [197] (Fig. 6). The hollow structure of nature-derived self-assembly supramolecular proteins are also suitable for internal modification for delivery of chemicals or other protein delivery, such as GFP [198], HIV protease [199], daunomycin [192] carboplatin [191] and even show possibility of gene delivery [200] using a hollow supramolecular structure. For example. Uchida et al. synthesized human H-chain ferritin with iron oxide in the hollow and cancer cell-targeting peptide RGD-4C attached to the surface. This supramolecular showed increased cancer targeting ability without perturbation of the self-assembly of ferritin structure. [201] In addition, nanocage can be an effective drug delivery vehicle with a targeting function using the characteristic of self-assembled supramolecular at once [202].

Although the application of self-assembled hierarchical nanocages derived from nature also shows a high utility value, it is unsuitable for various applications because of the limited number of structural elements in nature. However, de novo designed nanocages have various controllable shapes, sizes, and high-order symmetries, providing different surfaces and structures for antigen valency and spacing. Different valency and spacing of the functionalization unit can enhance the effect of drugs by allowing the desired nanocage to be selected according to the characteristics of the target ligand distance or uptake optimal size [184]. The application of various symmetric multivalent antigens presenting designed nanocages as vaccines was developed by genetic conjugation between the N-terminus of the trimer subunit and the C-terminal of trimeric viral glycoproteins and improved immunogenicity. (Fig. 6B) [203, 204] In a different way, Divine et al. designed self-assembled nanocage with various symmetry using antibody-Fc binders as a building block. They demonstrated enhanced target avidity experimentally by activating Tie-2 pathway (Fig. 6C) and enhancing SARS-CoV-2 neutralization with self-assembled supramolecular structures with each targeting antibody [135]. Through this, the de novo supramolecular protein cage could serve a significant role in improving effectiveness of immune activity and drug delivery since they have optimal size and superior avidity, which can effectively treat cancer and virus-derived diseases suffering from low rates of drug delivery rates.

### Epitope recognition for immunology and diagnosis

Proteins are macromolecules that perform complex but essential tasks in living cells through the formation of protein clusters via self-assembly. For instance, naturally existing hemoproteins that can specifically recognize and associate with heme groups to capture oxygen molecules by their heme-prosthetic groups [205]. Moreover, the self-association of proteins or peptides, naturally existing or de novo designed, can be utilized to improve the diagnostic potential, especially in the detection of bacterial infection at the initial stage. Like, Liu et al. utilized the self-assembly of a rhodamine-modified peptide derivative for detecting bacterial infections in gram-positive bacteria [206]. For the same purpose, Qianet developed a method for sensitive and specific detection of infected phagocytic cells (Staphylococcus aureus), owing to molecular self-assembly [207]. Recently, Yang et al*. *developed a self-assembling peptide based probe for vancomycin in which a luminogen with aggregation-induced emission (AIEgen) was used as a responsive fluorescence turn-on motif [208]. Furthermore, the nanotubes composed of various self-assembled peptide have also been utilized for the detection of various bacterial pathogens, like *E. coli* [209] and other nitro-functionalized neurotoxins [210]. The self-assembly of another class of peptides, ion-complementary, has also been utilized for immobilization of various biomolecules, including enzymes and analytes. After its self-assembly, the peptides have outwardly oriented charged residues (K and E), which can be utilized for binding moieties such as glucose [211]. However, many researchers have utilized the self-assembly of proteins or peptides for the recognition of target species of concern. For instance, Bianchi et al. detected the oxidation of ammonia (sensitivity 2.83 L^−1^ cm^−2^) and urea (sensitivity 81.3 L^−1^ cm^−2^) through aligned deposition of peptide (diphenylalanine) microstructures onto thiolated gold electrodes [212]. Furthermore, Zhang et al*.* utilized the self-assembly of streptavidin protein to create arrays, facilitated by a DNA lattice, and utilized it for efficient protein detection. The developed system detects protein arrays (biotin) aided by rhodamine or other fluorescent probes [213]. Recently, Arai et al*.* developed a protein-sensing device from a self-organized glycopeptide bundle with glucose or galactose at the C-terminals. Because of this insertion, the glycopeptides rearranged to form a bundle that acted as an ion channel due to the interaction between the target protein (peanut lectin and concanavalin A) and the terminal sugar groups of the glycopeptides [214]. Furthermore, self-assembly has been utilized to diagnose the structural details of small proteins. Like, Liu et al. utilized protein engineering to create a self-assembled scaffolding system, making possible to see structural details of a small sized protein via cryo-EM. Assisted by a rigid alpha helical linker, they utilized the self-assembly of 17-KDa DARPin protein to create a cage with cubic symmetry. The resulting construct was analyzed via cryo-EM to explore the structural details of DARPin at near-atomic level (3.5-to 5-Å resolution) [215].

In addition, the self-assembled structures of peptides have been used to flexibly functionalize the devices for various biomolecular detections, avoiding complex lithography, and improving selectivity and performance. The functionalizations obtained from such self-assemblies are stable, both thermally and electrochemically. Like, *Ryu et.al* utilized the self-assembly of peptide building blocks into vertically aligned nanowires (thermally stable up to 200 °C) to enhance their capability for electrochemical detection of biomolecules. For this purpose, they utilized aromatic dipeptides such as diphenylalanine in aqueous solutions under ambient conditions [216]. The same class of peptides has been utilized for developing organic field effect transistors (O-FETs) for electrical measurements in which a self-assembled peptide layer was used as a dielectric in FET to sustain electric fields [217]. A similar approach was utilized by Gupta et al. as they developed the self-assembly of di-peptide based building blocks into ordered nanotubes with expanding stability over a wide range of pH [172].

### Supramolecular assembly in enzymatic function

**Fig. 7:**
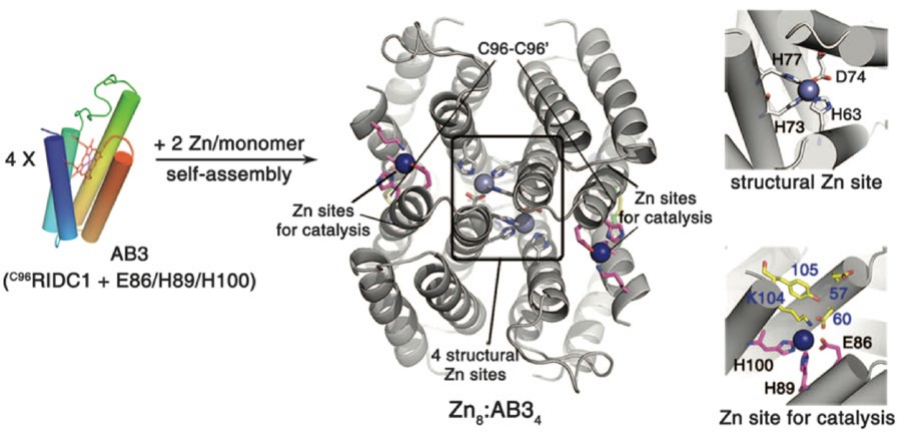
Example of enzymatic supramolecular structure via metal coordinate interaction (Image reprinted with permission from Ref [34])

In fact, ribosomal proteins (r-proteins) self-assemble to produce large protein content in cells by catalyzing ribosome production, which drives this process. This nature-derived catalytic process helps ribosomes to reduce the time required to produce a new set of r-proteins, facilitating cell growth [218]. Inspired by this nature-derived self-assembly process for catalyzing cell growth, researchers have utilized the self-assembly of designer proteins or peptides to create catalytic sites [35, 219], especially metallo enzyme [220–222], which can efficiently tune the properties of a metal ion to catalyze difficult chemical transformations (Fig. 7). Like, Woon et al. utilized the self-assembly of monomeric redox protein already possessing catalytic zinc sites at its interfaces, to design an artificial metallo-β-lactamase enzyme. The designed catalyst was not only functional in the periplasm of gram negative bacteria (*Escherichia coli*), enabling them to survive in the presence of antibiotic like ampicillin, but also displayed catalytic proficiency for ampicillin hydrolysis [223]. The designed metalloenzyme has also been utilized to catalyze oxidation reactions. As Olga et al. designed the supramolecular self-assembly of a peptide assisted by copper, for efficient catalysis of oxidation of dimethoxyphenol in the presence of dioxygen [224]. In the recent Lee et al., have also contributed to the work by improving the efficiency of the oxygen reduction reaction (ORR) by designing a self-assembly (Hexcoil-Ala) peptide. The designed peptide readily assembles on single-walled carbon nanotubes and helps in dispersion in aqueous solutions. Moreover, through mutation of the cysteine residue, a size-controlled and well-dispersed arrangement of AuNPs around the designed peptide has made possible which in turns gives improved electronic properties for enhanced oxygen reduction reaction performance in fuel cells [225]. Meanwhile, another group of scientists utilized the self-assembly property of designed collagen-mimetic peptides (CMPs) to create a supramolecular structure. They improved the self-assembly property of CMPs via metal-histidine coordination method and utilized the resultant superstructure for to catalyze ester hydrolysis in the presence of Zn(II) ions [226]. Recently, Hyun et al*.* designed a metallo-catalyst for efficient and industrially applicable biological conversion of methane to methanol. For the purpose, they reassemble the native catalytic domains of an already existing enzyme (methane monooxygenase). Through their construct, they not only successfully synthesize a stable and soluble protein construct in *Escherichia coli*, but also improved the yield of methanol while retaining enzymatic activity [227]. In addition, another group of scientists utilized the self-assembly of naturally existing proteins to create catalytically active sites for their application in catalysis. Like, Rubinov et al*.* utilized the self-assembly of amphiphilic peptides to form various well-defined structures like β-sheets, β-plates, fibrils and nanotubes, etc. Through these structures, they synthesized monomeric peptides, starting from basic building blocks [228]. To this end, a group of scientists explored another pathway of utilizing the self-assembly of two structurally different proteins (for instance Bovine Serum Albumin and bacterial microcompartment domain protein, PduBB), which do not possess any catalytic activity individually. However, upon self-assembly, a floral nanohybrid is formed which can catalyze the oxidation of pyrogallol to purpurogallin [229]. Despite of the way, many researchers have utilized the self-assembly phenomena of designer proteins to enhance the catalytic activity of various enzymes. Like, Zhang et al. designed an artificial hydrolase via self-assembled peptide nanofibers as biological enzymes for catalyzing ester hydrolysis [219]. Also, Wang et al. utilized the self-assembly of de novo designed helical hepta-peptides to create a phosphate mimic. The hydrolysis efficiency of these designer catalysts is comparable to already available enzymes like adenosine triphosphatase (ATPase) and alkaline phosphatase (ALPase), and hence can serve as a substitute [230]. Meanwhile, another group of scientists utilized naturally existing proteins to enhance their native catalytic activity via self-assembly. Like, Soares et al. explored the self-assembly of lipopeptides in water and utilized them for catalysis (yield was raised to 499% with selectivity up to 85%) of aldol reaction, using cyclohexanone and *p*-nitrobenzaldehyde as reactants [231]. In addition, Shan et al*.* utilized self-assembled films made from three distinct proteins (chitosan, laponite, and hemoglobin) for their application in electrochemistry and catalysis for hydrogen peroxide production. The film exhibited long-term stability, while the cyclic voltammetry peak potentials remained unchanged, and the cathodic peak currents remained undeclined even after 60 days [232].

### Protein design in the unprecedented material

**Fig. 8:**
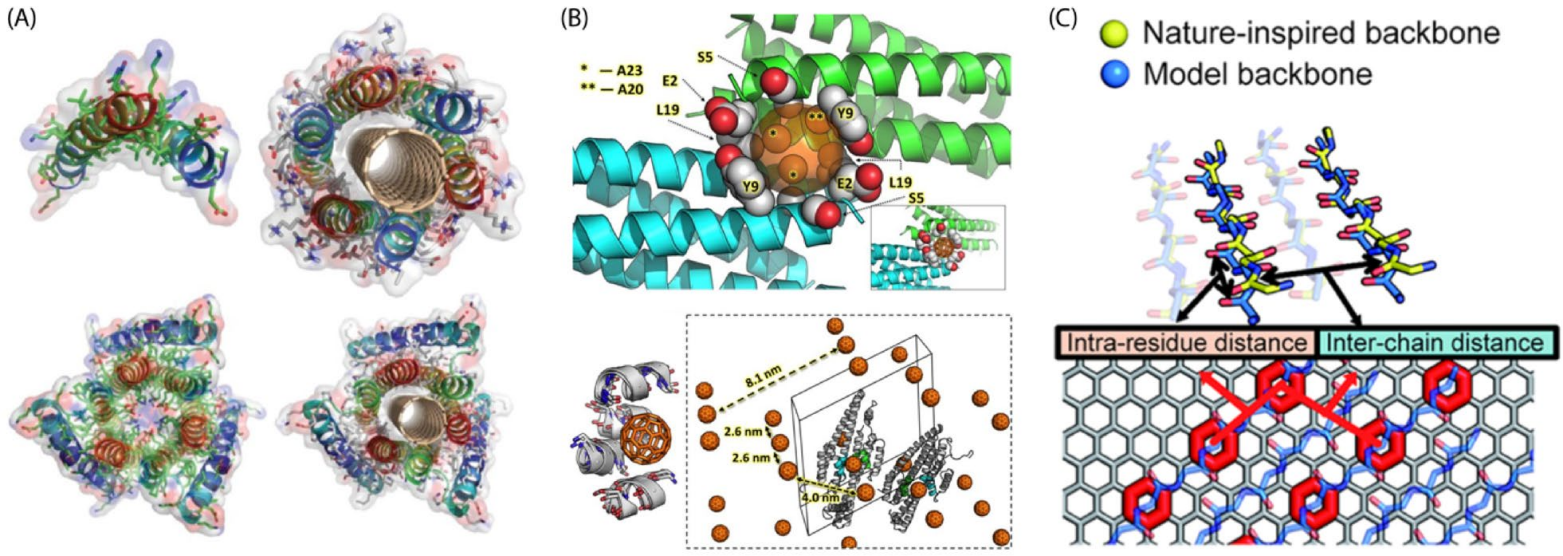
Example of supramolecular protein assembly with carbon material **A** single-wall carbon nanotube, **B** C60 fullerene, and **C** pristine graphene (Image reprinted with permission from Ref [240, 243, 248])

Inspired by the binding of amphipathic peptides with natural carbon materials such as graphite [233, 234], de novo designed peptides have been developed to induce self-assembled 2D structures on the surface of various carbon materials, such as graphene [235–237] or graphite (Fig. 8) [238, 239]. Remarkably, Grigoryan et al*.* designed peptides having capability of creating well textured surface by wrapping single-walled carbon nanotubes (SWNTs) in a structurally specific manner, creating a richly textured molecular surface. For binding with SWNTs, despite selecting peptides by phage-display or synthesizing peptides favoring SWNT binding, they developed a new design rule by utilizing a computational approach with an intrinsic recognition motif. They achieved higher-order assembly and dense packing by placing Gly and Ala in a repeating manner on a helix as the elementary structural unit [240]. Ko et al. thus, synthesized peptides that can self-assemble onto carbon nanotubes, the gold–platinum (AuPt) bimetallic nanostructures of which can induce a catalytic response to oxygen reduction [241].Meanwhile, a group of scientists developed the designer proteins or peptides that can direct self-assembly to produce ordered structures. Garima et al. developed a self-assembled monolayer induced by human serum albumin protein, that can convert 2D polyethylene glycol into discrete ring structures [242]. Similarly, Kim et al. demonstrated that the formation of ordered superstructures of buckminsterfullerene (C60) can also be directed by proteins [243]. In addition, researchers have utilized the self-assembly properties of proteins or peptides to hybridize other 2-dimensional carbon-based materials, such as graphene [244–246]. In this regard, Mustata et al. designed peptides forming two-dimensional monolayer crystals via self-assembly, eventually forming long, parallel, in-register b-sheets [247]. Despite designing peptides to self-assemble on graphene or graphite surfaces, some researchers have targeted pristine graphene owing to its excellent electrical properties and absence of any kind of functional moieties. For instance, No et al. developed nature-inspired designer peptides through the optimization of peptide-peptide and peptide-graphene interactions. Further, through the simulation, followed by experimentation, they proved that the designer peptides can self-assemble on to pristine graphene [248].

## Conclusions

To date, utilizing protein self-assembly from nature to designed supramolecular interfaces has been widely developed and has been proven to be a powerful tool for various application. Recently, as protein folding prediction using artificial intelligence, such as Alphafold [249] and RoseTTAFold [250], and computational tools have become very sophisticated, it is possible to make folding units with more diverse structures. This enables the assembly of proteins with more complex structures and functions. Folding units with structural flexibility or allosteric properties are among the most fascinating research directions. With these noble folding units, many researchers have designed protein assemblies that have more clinical and therapeutic applications beyond academic curiosity. For example, while maintaining stability in the in vivo environment, the assembly pattern is changed, the drug contained in the structure is delivered into the body, or the ligand related to signal transduction and receptor signal transduction in the body is labeled on the surface of the supramolecule.

## Data Availability

The datasets used and analyzed during the current study are available from the corresponding references listed.
